# *Nematopsis* Schneider, 1892 in Nerite Gastropods From Saint Kitts, With a Phylogenetic Study of the Genus, and Placement Within the Phylum Apicomplexa Levine, 1970

**DOI:** 10.1111/jeu.70023

**Published:** 2025-06-24

**Authors:** Nicole A. M. Herbert, Árni Kristmundsson, Nuria Vazquez, Kelsey Hoag, Mark A. Freeman

**Affiliations:** ^1^ Center for Conservation Medicine and Ecosystem Health Ross University School of Veterinary Medicine Saint Kitts Saint Kitts and Nevis; ^2^ Caribaea Initiative Les Abymes Guadeloupe; ^3^ Institute for Experimental Pathology at Keldur University of Iceland Reykjavik Iceland; ^4^ Laboratorio de Parasitología (LAPA) Instituto de Biología de Organismos Marinos (IBIOMAR) (CCT CONICET‐CENPAT) (U9120ACF) Puerto Madryn Argentina; ^5^ Verandah Pet Hospital Ft. Myers Florida USA

**Keywords:** Cephaloidophoroidea, gastropod, gregarine, *Nematopsis*, nerite, Porosporidae

## Abstract

The Apicomplexa are obligate unicellular parasites found in terrestrial and aquatic environments. *Nematopsis* are found infecting marine invertebrates in a life cycle involving mollusks and crustaceans. In the present study, mantle and muscle tissues from nerite gastropods were microscopically examined for *Nematopsis* using wet mounts and histology. Oocysts contained one single sporozoite surrounded by an ellipsoidal wall and were surrounded by a parasitophorous vacuole within a host phagocyte. Length and width of fresh oocysts were measured and compared between host species. DNA was extracted from infected tissue, and regions of the rRNA gene were amplified using novel primers. *Nematopsis*‐infected tissues from scallops from Argentina and Scotland were used as controls. The sizes of oocysts observed in nerite hosts from Saint Kitts were not significantly different. DNA sequences of *Nematopsis* isolated from nerite hosts in this study were identical and phylogenetically related to sequences obtained from scallops. Bayesian and maximum likelihood phylogenetic analyses robustly place the *Nematopsis* DNA sequences from this study with *Nematopsis* from scallops in Florida and members of the family Porosporidae. We conclude that marine *Nematopsis* will group within this clade or within the Porosporidae. We have provided specific oligonucleotide primers to assist with the molecular study of the Porosporidae.

## 1 Introduction

Apicomplexans comprise a major group of unicellular eukaryotic alveolates and are found in obligate parasitic relationships with a wide range of organisms (Mathur et al. 2019). These parasites are found in terrestrial and aquatic animals and include medically and veterinary important groups like the Coccidia (e.g., *Eimeria*, *Neospora*, *Sarcocystis* and *Toxoplasma*) and the vector‐borne zoonotic *Hematozoa*, which include *Plasmodium* and *Babesia* (Bermudez et al. 2021; Boisard et al. 2022; Dubey 2004; Kawahara et al. 2010). More ancestral apicomplexans such as the Marosporida infect marine invertebrates and cause epizootics in valuable wild shellfish populations (Kristmundsson et al. 2015; Kristmundsson and Freeman 2018; Mathur et al. 2019; Pales Espinosa et al. 2023). Other deeper‐branching apicomplexans such as the gregarines infect a myriad of terrestrial and aquatic invertebrates. They are typically found in the intestinal tract but are not usually associated with disease (Rueckert et al. 2019). However, a recent report has implicated gregarines as the cause of a mass mortality event in freshwater mussels (Alfjorden et al. 2024).

*Nematopsis* are almost exclusively marine gregarine apicomplexan parasites that complete their heterozenous life cycle between mollusks and decapod crustaceans (Azevedo and Padovan 2004; Sprague and Orr 1955; Zainathan et al. 2022). Mollusks act as the intermediate host, where *Nematopsis* develops as oocysts within connective tissue, gills, mantle, and muscle (Azevedo and Cachola 1992; Erazo‐Pagador 2010; Fajer‐Ávila et al. 2005; Poulpanich and Withyachumnarnkul 2009; Sabry et al. 2011; Sprague and Orr 1955; Uddin et al. 2011). In the intermediate mollusk host, infestation of *Nematopsis* presents as a variable number of oocysts encased within a parasitophorous vacuole. Each oocyst contains a single sporozoite and is encased within a wall (Tuntiwaranuruk et al. 2004). The vegetative stages, or gametocysts, of the parasite, are found in the gut epithelium of crustacea, which serves as the definitive host (Tuntiwaranuruk et al. 2015).

*Nematopsis* are important parasites negatively impacting several economically valuable marine mollusk and crustacean species worldwide (Azevedo and Cachola 1992; Canestri‐Trotti et al. 2000; Fajer‐Ávila et al. 2005; Özer and Güneydağ 2014; Sprague and Orr 1955; Poulpanich and Withyachumnarnkul 2009; Suja et al. 2016). The molluscan host is typically restricted to a range of bivalve taxa; however, *Nematopsis gigas* was isolated and described from a nerite gastropod, *Nerita ascencionis*, in Brazil (Azevedo and Padovan 2004), and *Nematopsis* has also been reported infecting a chiton (Lauckner 1983). *Nematopsis gigas* was characterized using traditional techniques such as light and electron microscopy, with the morphology and ultrastructure of the parasite being described from the gastropod. The definitive host remains unknown (Azevedo and Padovan 2004).

More recently, DNA sequence data has become available for the previously described freshwater gregarine, *Nematopsis temporariae*, found infecting the liver of the tadpole stage of the European common frog, *Rana temporaria* (Chambouvet et al. 2016; Nöller 1920) a freshwater vertebrate known to host other apicomplexan parasites (Jirku et al. 2009). In addition, recent molecular data have become available for a marine *Nematopsis* species observed histologically from the bay scallop (*Argopectin irradians*) in Florida (Scro et al. 2023). No other DNA data currently exist for marine *Nematopsis* spp. infecting other mollusk or crustacean hosts. Molecular data is, however, available for the genus *Porospora* within the same family Porosporidae, *Porospora gigantea* (Boisard et al. 2022), which was isolated from the gut lumen of the European lobster, *Homarus gammarus* .

The aims of this research were to report a *Nematopsis* parasite infecting nerite gastropods and to provide DNA sequence data for marine *Nematopsis* with the description of novel oligonucleotide primers that will facilitate further study within the family Porosporidae. This study will also provide *Nematopsis* DNA data amplified from scallop hosts in Scotland and Argentina to help place marine *Nematopsis* in a phylogenetic context with *Porospora* and, more broadly, within the Apicomplexa. We hypothesize that marine *Nematopsis* and the Porosporidae in general will be genetically distant from *Nematopsis temporariae* from a freshwater vertebrate, which likely belongs to a different family within the gregarines.

## 2 Materials and Methods

### 2.1 Sample Collection and Microscopy

Gastropods were collected from two rocky shorelines of Saint Kitts, namely Ross University School of Veterinary Medicine (17.2951°N, −62.7621°W), located on the Western side of the Island and Gong Beach (located on the Eastern side of the island's peninsula; 17.2509°N, −62.6437°W) from July 2022 to July 2023. Individuals were placed in seawater during transport to the laboratory, where they were cooled in a refrigerator, removed from the shell, and dissected. A total of 117 bleeding tooth nerites, *Nerita peloronta* (Ross University School of Veterinary Medicine: RUSVM) (*n* = 56); Gong Beach (*n* = 61), and ten *Nerita versicolor* (RUSVM), were included in this study. Photos were taken of the ventral and dorsal planes of each species for identification purposes. Specimens were dissected, and sections of the mantle and foot muscle were removed and examined using a compound microscope. Images of the infected mantle and foot were taken, where oocysts were photographed, and measurements were taken using CellSens imaging software. The sizes of oocysts were compared using a T‐test. Infected tissue was collected and fixed in 2% sodium chloride in buffered 10% formalin for histological analysis and also stored in lysis buffer for later molecular analyses. Fixed samples were processed using standard methods, sectioned at 4 μm and stained with hematoxylin and eosin. Specimens of other gastropod species were also collected and analyzed for the presence of the parasite. These gastropods were also identified using morphology and molecular techniques.

### 2.2 DNA Extraction From Gastropods

Infected tissue samples (approximately 25 mg) were placed in lysis buffer (from the GeneMATRIX Tissue DNA purification kit) until DNA extractions could be undertaken and PCR performed. DNA samples of scallop tissues infected with *Nematopsis* extracted from *Aequipecten tehuelchus* (Tehuelche scallop) from Argentina and *Pecten maximus* (King scallop) from Scotland, identified as *Nematopsis*‐infected by microscopy, were included in this molecular study.

Total DNA was extracted from all gastropod and scallop tissue using a DNA extraction kit (GeneMATRIX Tissue DNA purification kit), following the tissue protocol. For species identity confirmation some snails were identified with barcoding methods, where the ‘Folmer’ primers LCO1490 (5′ ggtcaacaaatcataaagatattgg 3′) and HCO2198 (5′ taaacttcagggtgaccaaaaaatca 3′) were used to amplify the mitochondrial cytochrome oxidase subunit 1 (COI) region (Folmer et al. 1994). The majority of PCR reactions in this study were performed at a 20 μL total volume, were comprised of 1.5 μL of each primer (5 U), 0.2 μL Taq polymerase, 2 μL 10× buffer, 2 μL dNTP (0.25 mM). Some PCR reactions were performed in 25 μL total volume comprising 1 μL DNA template and 45 nM of each primer and a 1× concentration of TaqDog Premium 2× Green Master Mix.

### 2.3 Molecular Analysis

Efforts were made to amplify *Nematopsis* DNA using existing primers designed for aquatic apicomplexans of invertebrates (Kristmundsson et al. 2011; Kristmundsson et al. 2015). However, insufficient amplification necessitated the design of more specific primers targeting members of the genera *Porospora* and marine *Nematopsis*. The DNA sequences of *Porospora gigantea* (Accession numbers OM811444 and OM811445) and those of other closely related apicomplexans were downloaded from NCBI and aligned in ClustalX software.

These alignments served as a reference point for the design of primers that amplify the small subunit ribosomal DNA (SSU rDNA) sequence of the parasite. The resulting primers, 18e‐Nem (forward primer: 5′ ccggttgactctgccggt 3′) and Nem‐510‐rev (reverse primer: 5′ cctcagatcaatagcgag 3′), primer pair Poro120fwd (5′ atacgtcgaaaccgcagac 3′) and Poro990rev (5′ tggctgttgagtacgagtg 3′) and primer pair Nem510fwd (5′ ctcgctattgatctgagg 3′) and 18gMn‐rev (5′ tcaacaactgttgccgtg 3′) were used on all samples (Table 1). The individual reads, both forward and reverse, generated from these three primer pairs were used to create consensus sequences for the SSU rDNA. In addition, other primers were designed to amplify the ITS1, 5.8 s, and ITS2 region of the parasite rDNA. The ITS region was amplified using the Poro‐18gMn‐fwd (5′ agtcgtaacacggcaacag 3′), Poro1357‐fwd (5′ taacgaacgagatctcgac 3′) and Poro‐ITS2‐rev (5′ gtaatatgcttacgttcg 3′) primers (Table 1). All PCR reactions were conducted using the following conditions and performed using a Flexilid Mastercycler (Eppendorf, Hamburg, Germany): initial denaturation at a temperature of 95°C for 10 min, then 40 cycles of denaturation at 94°C for 30 s, annealing at 55°C for 1 min, and extension at 72°C for 1 min. The final step of these reactions was a final extension at 72°C for 7 min.

**TABLE 1:** Nucleotide sequences, target region, and approximate amplicon size of novel primers used in this study, designed to be broadly specific for the Porosporidae.

Primer name	Target region	Primer sequences (5′ to 3′)	Amplicon size (bp)
18S (SSU)	18e‐Nem	ccggttgactctgccggt	500
	Nem‐510‐rev	cctcagatcaatagcgag	
18S (SSU)	Nem510fwd	ctcgctattgatctgagg	1300
	18gMn‐rev	tcaacaactgttgccgtg	
18S (SSU)	Poro‐120‐fwd	atacgtcgaaaccgcagac	700
	Poro‐990‐rev	tggctgttgagtacgagtg	
ITS	Poro‐18gMn‐fwd	agtcgtaacacggcaacag	600
	Poro‐ITS2‐rev	gtaatatgcttacgttcg	
SSU and ITS	Poro‐1357fwd	taacgaacgagatctcgac	800
	Poro‐ITS2‐rev	gtaatatgcttacgttcg	

All PCR products were column purified (GeneMATRIX PCR/DNA Clean‐Up purification kit) and sent to an external company for bidirectional sequencing using the same primers. The quality of each sequence was checked by examining the chromatogram viewed using BioEdit, and nucleotide BLAST searches were performed on each sequence to either check for apicomplexan similarity or to identify/confirm the species of snails in the study. Contiguous sequences were compiled manually using ClustaX and BioEdit software.

### 2.4 Phylogenetic Analyses

Phylogenetic analyses were performed on a ClustalX‐created alignment of 47 apicomplexan taxa consisting of 1910 informative sites. Maximum likelihood analyses were run using the general time‐reversible substitution model (GTR) and 100 bootstrap repeats on PhyML (Guindon et al. 2010). Bayesian inference was performed on MrBayes v3.2.7 (Ronquist and Huelsenbeck 2003); posterior probability distributions were generated using the Markov Chain Monte Carlo (MCMC) method, with four chains being run simultaneously for 2,000,000 generations. Burn‐in was set at 2500 and trees were sampled every 100 generations to compile the majority rule consensus trees. All phylogenetic trees were viewed in FigTree v1.4.4 (Rambaut 2018) and annotated in Adobe Illustrator.

## 3 Results

**FIGURE 1:**
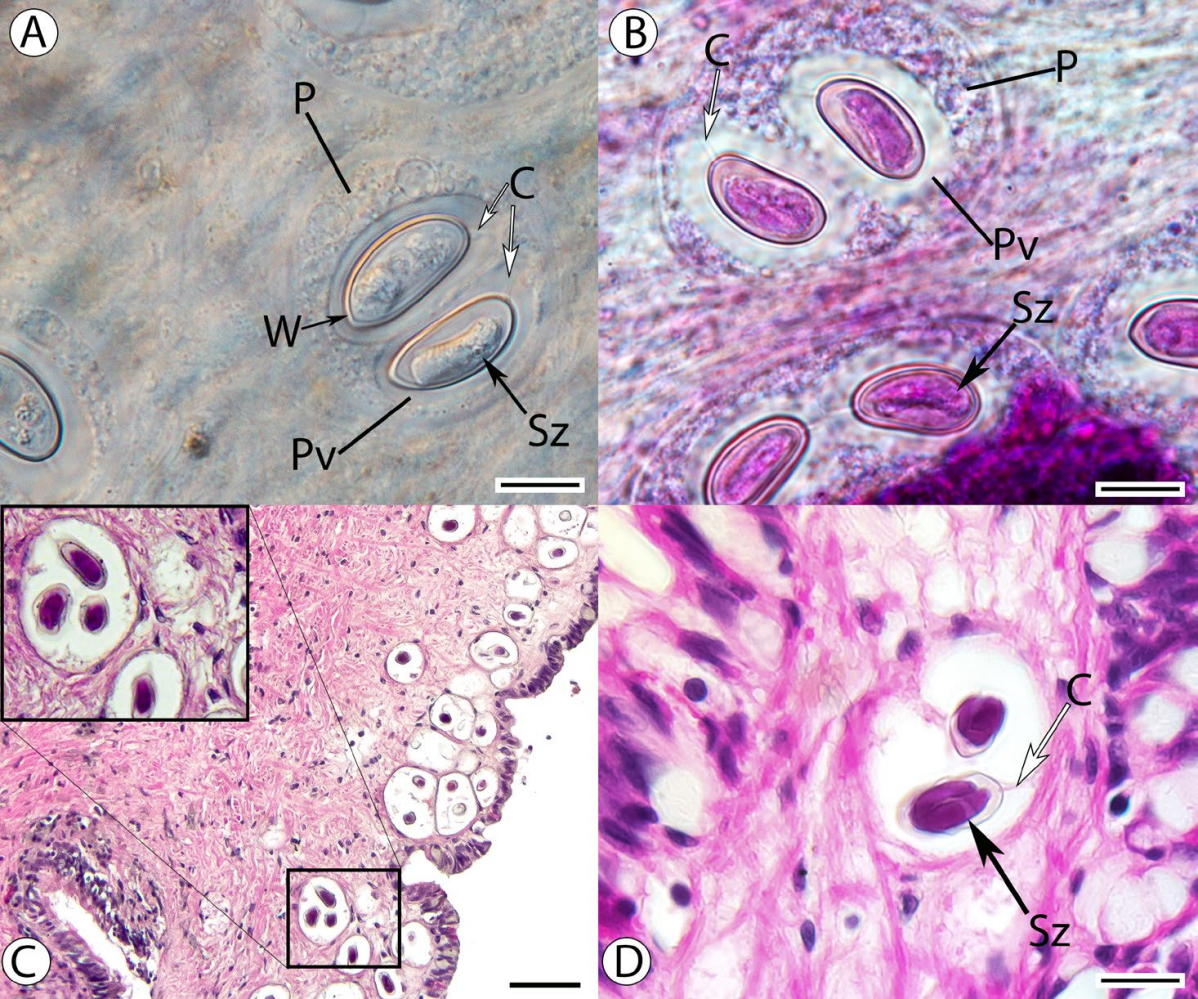
Wet mounts and histomicrographs of *Nematopsis* observed in nerite gastropods from Saint Kitts. A. Wet mount and B. Gram‐stained wet mount observed using Differential Interference Contrast (DIC) showing oocysts surrounded by a thick wall (W) within a parasitophorous vacuole (Pv), all within the host's phagocyte (P). Each oocyst contains one sporozoite (Sz). The apical ends of the oocyst walls (location of the operculum) are attached to the parasitophorous vacuole via a cord (C). C. and D. Histomicrographs showing oocysts within a phagocyte, with oocyst, cord (C) and sporozoite (Sz) as clearly seen structures within the host tissue. The apical end of the oocyst walls is attached to the parasitophorous vacuole via a cord. This apical end is also covered by an operculum. Scale bars: A, B = 11 μm; C = 40 μm; D = 10 μm.

All nerite snails were identified by morphology and by barcoding as either *N. peloronta* or *N. versicolor* . Of the 117 *N. peloronta* specimens examined microscopically, 92 were infected with *Nematopsis* (78.6%) and *Nematopsis* was observed in 10 of 10 (100%) of *N. versicolor* screened. All non‐nerite species, 37 individual snails comprising 4 species ( *Plicopurpura patula* (*n* = 4), *Echinolittorina ziczac* (*n* = 14), *Supplanaxis nancyae* (*n* = 9) and *Tegula* sp. (*n* = 10)) were not infected with *Nematopsis*, based on microscopic analysis. In *N. peloronta* , both microscopic and histological analysis showed oocysts developing in parasitophorous vacuoles within a host phagocyte, structures typical of *Nematopsis* (Figure 1, ranging from one to eleven oocysts). A cord that connects the oocyst to the host phagocyte is also apparent. No associated pathology of host tissues was observed during histological analysis. Fresh oocysts (*n* = 39) observed in *N. peloronta* measured 17.57 (± 0.95) μm long (range: 16.50–19.20) and 8.88 (± 0.64) μm wide (range: 7.13–10.65) while those observed in *N. versicolor* (*n* = 30) were 17.83 (±0.47) μm long (range: 16.26–19.42 μm) and 8.73 (± 0.28) μm wide (range: 7.23–10.18 μm). The size of oocysts isolated from these two hosts was not significantly different (length: *p* = 0.227; width: *p* = 0.362).

The SSU rDNA consensus sequence of the Saint Kitts *Nematopsis* amplified by the three SSU primer pairs (representative of *Nematopsis* isolated from the nerites *N. peloronta* and *N. versicolor* ) was compared to those isolated and amplified from Scotland and Argentina. The Saint Kitts sequence and the *Nematopsis* from Scotland were 95.54% similar, while that from Argentina was 96.09% similar to Saint Kitts. Sequences from the pectinids from Argentina and Scotland were 97.77% similar (Table 2). Further to this, the ITS regions amplified by Poro‐18gMn‐fwd and PoroITS2‐rev, when compared, were as follows: Saint Kitts and Scotland were 86.1% similar, while Saint Kitts and Argentina were 83.5% similar. The regions amplified from pectinids from Scotland and Argentina were 93.9% similar (Table 2). Comparisons of both the SSU and ITS regions of *Nematopsis* isolated from both *N. peloronta* and *N. versicolor* revealed that the isolates were identical. The consensus sequences obtained in this study have been submitted to Genbank under the accession numbers PV470980‐2.

**TABLE 2:** Genetic similarity values (%) of *Nematopsis* isolated from Saint Kitts, Argentina, and Scotland, based on sequences of 18S region (values above diagonal; 18e‐Nem to 18gMn‐rev, approximately 1800 bp) and based on sequences of ITS region (Poro18gMn‐fwd to Poro‐ITS2‐rev, approximately 600 bp; values below diagonal).

	Saint Kitts	Argentina	Scotland
Saint Kitts	—	96.09	95.54
Argentina	83.5	—	97.77
Scotland	86.1	93.9	—

**FIGURE 2:**
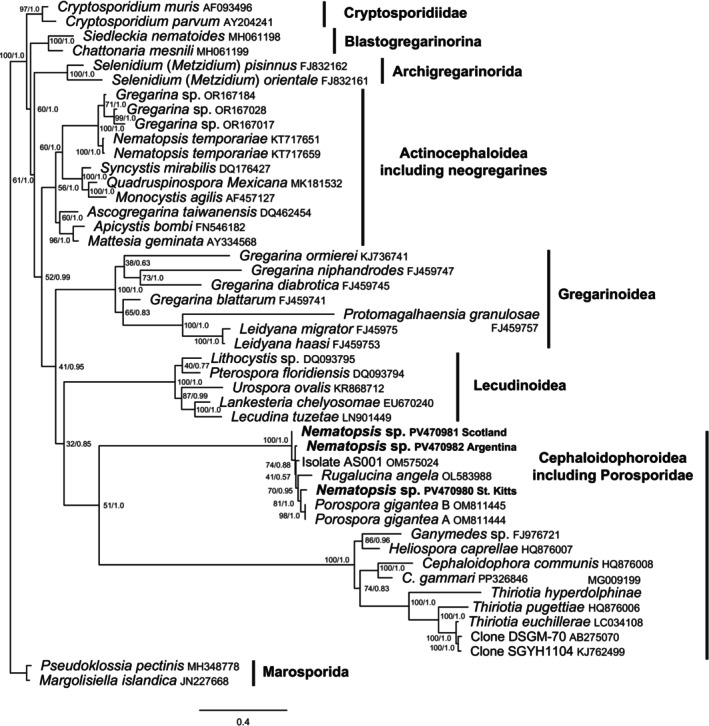
Maximum likelihood topology of 45 gregarine taxa using the Marosporida as the outgroup. Numbers at the nodes indicate bootstrap support values and posterior probabilities. The sequences generated in this study, for *Nematopsis* spp., are shown in bold and are fully supported in a clade with other members of the Porosporidae, which forms a moderately supported sister clade to members of the superfamily Cephaloidophoroidea. Family‐level or broader classifications, according to Simdyanov et al. (2017) and Park et al. (2025) are given on the right.

The nerite *Nematopsis* sp. from this study and the *Nematopsis* spp. isolated from scallops in Scotland and Argentina are all robustly phylogenetically placed with *Porospora gigantea*, another member of the Porosporidae for which sequence information is available (Figure 2). In addition, sequences downloaded from NCBI grouped in the same clade, namely those of an undescribed *Nematopsis* sp. isolated from the bay scallop ( *Argopecten irradians* ) in Florida and a sequence labeled as *Rugalucina angela*, which is a mollusk from Thailand. This fully supported clade, with other members of the Porosporidae, forms a moderately supported sister clade with other members of the superfamily Cephaloidophoroidea.

*Nematopsis temporariae*, a freshwater species, does not group with other members of the family Porosporidae but is instead more closely related to other freshwater gregarines.

## 4 Discussion

We report the presence of the marine gregarine *Nematopsis* in nerite gastropods from the Eastern Caribbean region of the West Indies and support these findings with molecular data and phylogenetic placement within the gregarines/Apicomplexa. In a survey of several mollusk species on Saint Kitts (Hoag 2019), *Nematopsis* sp. was reported in the bleeding tooth nerite, *Nerita peloronta*, while this current study reports *Nematopsis* in a second nerite species, *Nerita versicolor* . Historically, studies published more recently (Silva et al. 2019) have identified *Nematopsis* based on morphology, ultrastructure, and host species. However, studies on other oocyst and spore‐forming parasites such as eimerids and myxosporeans indicate that morphological features alone can be very limiting. Further to this, the molecular analysis provides valuable information for discriminating species and resolves phylogenetic relationships with related taxa (Jirku et al. 2009; Freeman and Kristmundsson 2018). Phylogenetic analyses have established the nerite parasite isolated in this study as a *Nematopsis* species, robustly grouping it with another genus, *Porospora*, in the family Porosporida*e*. Therefore, based on both the morphological and phylogenetic data of the parasite isolated from marine nerite gastropods on Saint Kitts, we conclude that it belongs to the genus *Nematopsis*.

*Nematopsis* is typically reported as a marine parasite infecting two invertebrates, mollusks and crustaceans. However, morphologically similar gregarines such as *N. temporariae* have also been placed in the genus *Nematopsis* (Chambouvet et al. 2016; Nöller 1920). The known hosts for *N. temporariae* are freshwater vertebrates, which do not align with the current understanding of *Nematopsis* spp., which are almost exclusively described as infecting marine invertebrates. From our phylogenetic tree (Figure 2), *N. temporariae* is too distant from other members of Porosporidae to be consideredpart of the family, considering that identification and characterization of *Nematopsis* spp. is traditionally based on the analysis and description of oocysts and trophozoites in marine mollusks and crustaceans (Suja et al. 2016; Azevedo and Matos 1999; Chakraborti and Bandyopadhyay 2010). Recent phylogenetic analyses conducted on apicomplexan parasites of the freshwater mussel *Margaritifera margaritifera* support this finding. Authors stated that the phylogenetic placement of *N. temporariae* in a terrestrial clade does not align with what is typical regarding host and habitat and molecular phylogenetics and may not truly be a member of the genus (Alfjorden et al. 2024). Other researchers question the inclusion of *N. temporariae* within the *Nematopsis* genus since the life cycle is not typical, and morphologically, oocysts do not possess structures usually present in *Nematopsis* spp., such as an operculum (Azevedo et al. 2023). It is also interesting to note that our phylogenetic analyses (Figure 2) place members of the genera *Nematopsis*, *Porospora*, *Ganymedes*, *Heliospora*, *Cephaloidophoea*, and *Thiriotia* in the same clade, Cephaloidophoroidea, a superfamily. This occurrence aligns with those previously reported, which notes all members of this clade are eugregarine parasites found in the intestine of marine crustacean hosts (Diakin et al. 2016; Simdyanov et al. 2017).

It may be valuable to identify the intermediate host of *N. temporariae* to properly elucidate the characteristics of this species. Combining this along with further phylogenetic analysis will result in a clear characterization of the genus and its correct positioning within the Apicomplexa. Further to this, a *Nematopsis* sp. has been reported in the bay scallop, *Argopectin irradians*, in Florida using metagenomic analyses (Scro et al. 2023). The 571 bp‐long sequence of the SSU rDNA region (OM575024) included in our phylogenetic analyses (Figure 2) lends additional support for the placement of the apicomplexan isolated in Saint Kitts in the genus *Nematopsis*. Sequences from *Nematopsis* isolated from scallops in Argentina and Scotland also give further support for the placement of the parasite isolated in Saint Kitts as a member of *Nematopsis*. This study is the first to phylogenetically place *Nematopsis* spp. sourced from varied geographical locations within the Porosporidae (Apicomplexa).

This study has also expanded the DNA sequence data for the Porosporidae, revealing unusual regions of conserved nucleotide sequences within the family, compared to other apicomplexans, which have facilitated the construction of family‐specific primers (Table 1) to assist with future research into this intriguing group of marine parasites.

The genetic sequences of *Nematopsis* spp. isolated from scallops from temperate locations, Scotland and Argentina, showed a relatively high percentage identity based on the entire SSU region (approximately 98%) and the ITS region (approximately 94%), considering their geographical distance. This suggests that there is limited genetic drift over long periods of time in these parasites and limited host switching, indicating that a degree of host specificity is likely to be a characteristic of these parasites. Other researchers have seemingly inadvertently amplified *Nematopsis* from a mollusk in Thailand (OL583988). This study provides PCR primers that can be used in other regions of the world for screening and further study of *Nematopsis*. The primer pairs detailed in Table 1 can be used to serve as diagnostic or environmental primers for marine *Nematopsis* and *Porospora*, allowing for screening of potential intermediate and definitive hosts for infection without host or other parasite amplification.

*Nerita peloronta* and *Nerita versicolor* are new nerite hosts for *Nematopsis*, which is typically found in bivalve molluscan hosts such as scallops, oysters, and mussels (Azevedo and Matos 1999; Silva et al. 2019; Suja et al. 2016). Only one additional report of *Nematopsis* exists in a nerite gastropod, *Nematopsis gigas*, isolated from the mantle of *Nerita ascensionis*, found on the Atlantic Coast of Brazil (Azevedo and Padovan 2004). Oocyte comparisons of specimens collected from *N. ascensionis* (21.9 ± 0.5 μm long and 11.5 ± 0.6 μm wide) and *N. peloronta* (17.57 ± 0.95 μm long and 8.88 ± 0.64 μm wide) reveal a significant difference in size compared to the *Nematopsis* from nerites in Saint Kitts. Although *Nematopsis* was isolated from two nerite species in Saint Kitts, it was determined, using morphological and molecular analysis, that the parasite isolates were identical. Therefore, we report here one *Nematopsis* sp. in two nerite species, *Nerita peloronta* and *N. versicolor* . Our initial assumption was that the *Nematopsis* sp. in this study possessed a narrow host range, infecting only one molluscan host species, the bleeding tooth nerite. But due to our findings, we conclude that the *Nematopsis* reported from Saint Kitts may be specific to closely related members of the genus *Nerita*. *Nematopsis gigas* has been reported infecting one nerite gastropod species in Brazil (Fernando Noronha Island), which is in the Atlantic Ocean approximately 4000 km from Saint Kitts. There is a possibility that the parasite isolated in this study may be *N. gigas* ; however, given the geographical distance and the statistically significant difference in oocyst size, we consider this to be unlikely. However, if possible, molecular analyses should be conducted on *N. gigas* , and the sequences should be compared with those generated in this study before a new species is described.

Although detected in more than one nerite species in Saint Kitts, *Nematopsis* was not detected in gastropods belonging to other genera examined in this study and that conducted by Hoag (2019). This suggests that gastropods are likely not typical molluscan hosts and that nerites may be an exception as the only gastropod intermediate host of *Nematopsis* in the region. Future studies in the region should explore the presence and prevalence of infection in bivalves and crustaceans.

In a study in Thailand, where seven species of bivalve were screened for infection, four different oocyst morphotypes were reported. Although each morphotype was not identified to a species level, there is also a possibility of a relatively broad host range, where one *Nematopsis* species infects more than one genus of bivalve (Tuntiwaranuruk et al. 2004). More extensive molecular screening should be undertaken on a wider range of mollusks to determine the host range of these parasites, particularly of bivalves, and whether oocyst morphology is a good indicator for species discrimination.

While nerites may not be a focus of local Caribbean fisheries, these gastropods form part of an important tropical ecosystem. In addition, there are important mollusks, such as the West Indian top snail, *Cittarium pica* , and the Queen conch, *Strombus gigas* , which are valuable species in the Caribbean. Therefore, screening these and other marine mollusks is important from an ecological and food security standpoint. Further work can therefore include screening these species and other mollusks such as bivalves for the presence of *Nematopsis*. The definitive host of *Nematopsis* spp. is usually a decapod crustacean (Azevedo and Padovan 2004; Sprague and Orr 1955). Most studies report either the intermediate or the crustacean host, but rarely both hosts. Further studies should include identifying the crustacean host of the *Nematopsis* sp. found on Saint Kitts. Although the crustacean definitive host in this study is unknown, many *Nematopsis* spp. infect economically important crustaceans such as *Penaeus monodon* (black tiger shrimp) and *Litopenaeus vannemi* (the Pacific white shrimp) (Jiménez et al. 2002; Poulpanich and Withyachumnarnkul 2009). There are economically important crustacean species present in Saint Kitts, the most important of these being the Caribbean spiny lobster *Panulirus argus* ). While preliminary examination of the gut lumen of this species has not resulted in observation of the parasite, future research should include larger sample sizes, including adults and juveniles, of this and other crustacean species (Atherley et al. 2020).

We report the presence of a *Nematopsis* sp. in two nerite species, *N. peloronta* and *N. versicolor* , on Saint Kitts. The parasite was not found in other gastropods screened. Based on morphological analysis, this species may be different from that reported in *Nerita ascencionis* in Brazil. A comparison of the genetic sequences of *Nematopsis* in Saint Kitts and scallops in Argentina, Scotland, and Florida supports the placement of the parasite in Saint Kitts in the genus *Nematopsis*. Phylogenetic analyses also reveal that *Nematopsis temporariae* most likely does not belong to the genus *Nematopsis*.

## Conflicts of Interest

The authors declare no conflicts of interest.

## Data Availability

Research data are not shared.
